# Impacts of environmental factors on fine root lifespan

**DOI:** 10.3389/fpls.2014.00205

**Published:** 2014-05-16

**Authors:** M. Luke McCormack, Dali Guo

**Affiliations:** Key Laboratory of Ecosystem Network Observation and Modeling, Synthesis Research Center of Chinese Ecosystem Research Network, Institute of Geographic Sciences and Natural Resources Research, Chinese Academy of SciencesBeijing, China

**Keywords:** ecosystem, root longevity, belowground, priming, nitrogen, phosphorus, climate change, mycorrhizal fungi

## Abstract

The lifespan of fast-cycling roots is a critical parameter determining a large flux of plant carbon into soil through root turnover and is a biological feature regulating the capacity of a plant to capture soil water and nutrients via root-age-related physiological processes. While the importance of root lifespan to whole-plant and ecosystem processes is increasingly recognized, robust descriptions of this dynamic process and its response to changes in climatic and edaphic factors are lacking. Here we synthesize available information and propose testable hypotheses using conceptual models to describe how changes in temperature, water, nitrogen (N), and phosphorus (P) availability impact fine root lifespan within a species. Each model is based on intrinsic responses including root physiological activity and alteration of carbohydrate allocation at the whole-plant level as well as extrinsic factors including mycorrhizal fungi and pressure from pathogens, herbivores, and other microbes. Simplifying interactions among these factors, we propose three general principles describing fine root responses to complex environmental gradients. First, increases in a factor that strongly constrains plant growth (temperature, water, N, or P) should result in increased fine root lifespan. Second, increases in a factor that exceeds plant demand or tolerance should result in decreased lifespan. Third, as multiple factors interact fine root responses should be determined by the most dominant factor controlling plant growth. Moving forward, field experiments should determine which types of species (e.g., coarse vs. fine rooted, obligate vs. facultative mycotrophs) will express greater plasticity in response to environmental gradients while ecosystem models may begin to incorporate more detailed descriptions of root lifespan and turnover. Together these efforts will improve quantitative understanding of root dynamics and help to identify areas where future research should be focused.

## INTRODUCTION

Describing and predicting patterns of plant traits and growth strategies has long attracted attention from plant and ecosystem ecologists (Grime, 1977; Reich et al., 1997). While advances in theoretical and empirical understanding has emerged for stems and leaves (Westoby and Wright, 2006; Chave et al., 2009), few cohesive theories or predictions are available for the growth and function of roots (Robinson et al., 2003; Kong et al., in press). This has limited empirical understanding and modeling of key belowground processes (Ostle et al., 2009; Iversen, 2010). In particular, processes that occur through time and require repeated observations such as fine root lifespan and turnover have been difficult to quantify despite their key roles in understanding and managing ecosystems.

The lifespan of fast-cycling, absorptive roots determines root turnover, and consequently impacts carbon (C), nutrient, and water cycles for plants and whole-ecosystems. While estimates vary, between 10 and 40% of net primary productivity is likely allocated to the production and turnover of fine roots (Jackson et al., 1997; Gaudinski et al., 2010), with much higher estimates reported in grasslands and higher latitude systems (Ruess et al., 2003; Hui and Jackson, 2006; Xu et al., 2012). This represents a major flux of ecosystem C into soils where root and root-associated C plays a disproportionately large role in priming microbial communities and building soil C stores (Drake et al., 2011; Tefs and Gleixner, 2012; Clemmensen et al., 2013). Additionally, because resource uptake rates decline with root age (Bouma et al., 2001; Volder et al., 2005), lifespan also impacts water and nutrient uptake by mediating the age structure of a root population. However, a paucity of robust and methodologically consistent data limit broad understanding of the complex interactions between root dynamics and C, water, and nutrient cycling in different ecosystems (Hendricks et al., 2006; Guo et al., 2008a; Strand et al., 2008).

Based on current evidence, it is clear that root lifespan and turnover are highly variable in both space and time, with a wide range of estimates reported across biomes (Gill and Jackson, 2000; Peek, 2007), within biomes (Yuan and Chen, 2010), and across species within a single site (Coleman et al., 2000; Withington et al., 2006; McCormack et al., 2012). Importantly, patterns of root dynamics across species have recently been linked to root and whole-plant traits such that larger root diameters, higher root tissue density, lower root nitrogen (N) content, and slow whole-plant growth rate are associated with longer root lifespan and slow turnover (Ryser, 1996; Tjoelker et al., 2005; Withington et al., 2006; McCormack et al., 2012). Still, more work is needed to determine how relevant these patterns are across a broader range of species and outside of temperate biomes where the majority of previous research has been focused.

Despite indications of patterns describing root dynamics across species, there is still a tremendous amount of variation within species that remains largely unexplored. Within site, marked differences in root turnover rate for the same species across multiple years have been reported (Gill and Jackson, 2000; Iversen et al., 2008; McCormack et al., in press). Furthermore, observations of individual species across multiple sites have also revealed substantial within-species variation. For example, greater than twofold variation in root lifespan has been observed in both *Pinus palustris* and *Pinus taeda* across sites in the southeastern United States (King et al., 2002; Guo et al., 2008b; Pritchard et al., 2008; Espeleta et al., 2009; McCormack et al., 2010), and significant differences in fine root lifespan were found across a network of four sites dominated by *Acer saccharum* in Michigan, USA (Burton et al., 2000). Despite these case studies revealing substantial plasticity in root dynamics within a species, we lack a framework to determine how different edaphic and climatic factors impact fine root lifespan and turnover within and across sites.

### A FRAMEWORK FOR PREDICTING CHANGES IN FINE ROOT LIFESPAN ALONG ENVIRONMENTAL GRADIENTS

In this review, we examine how within-species variation in absorptive fine root lifespan responds to four key environmental factors: water availability (precipitation), soil moisture, temperature, N availability, and phosphorus (P) availability. First, we focus on *within*-species variation because much of the response to changes in climate over the next century will be driven by plasticity within species rather than by species replacements, particularly for environments dominated by long-lived plants such as trees (Zhu et al., 2012; Prasad et al., 2013). Additionally, many plant species can be found across wide climate envelops (e.g., *Acer rubrum*, *Pinus sylvestris*, and *Lolium perenne*; Critchfield and Little, 1966; Burns and Honkala, 1990; Watson et al., 2000) and single point estimates of root dynamics are unlikely to adequately capture behaviors across a species’ full range. In the context of this review, we define plasticity to represent variation across the natural range of that species. This primarily represents phenotypic plasticity but in case of species growing across wide climate gradients, genotypic plasticity may also be important. It does not, however, encompass root responses to environmental conditions outside a species natural range (e.g., a temperate species growing in a tropical climate or a desert).

Second, we primarily refer to root lifespan rather than root turnover because lifespan is a simple parameter that can be directly measured and its variation can be related to environmental factors. By contrast, variation in root turnover can be difficult to interpret as shifts in turnover result from various combinations of increasing or decreasing root production, standing biomass, and mortality. Lifespan can be used to estimate turnover (McCormack et al., in press) which allows a framework describing root lifespan to be more broadly applied to turnover as well. Generally, increases in lifespan are related to decreases in turnover rate and *vice versa*.

**FIGURE 1:**
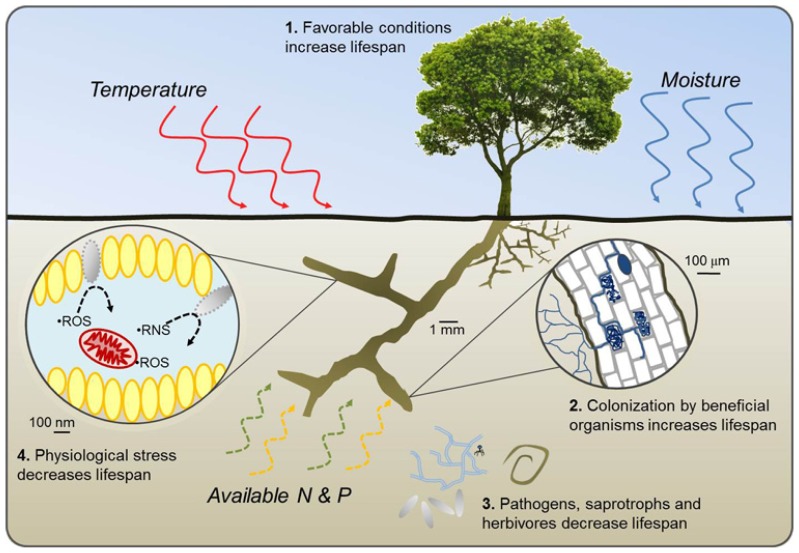
**Conceptual diagram of four pathways through which variation in temperature, water availability, and the availability of nitrogen and phosphorus either increase (1 and 2) or decrease (3 and 4) fine root lifespan.** *Pathway 1* represents a potential increase in lifespan with increased plant productivity, carbohydrate synthesis, and subsequent allocation of resources to fine roots under favorable growing conditions. In *Pathway 2*, colonization by some mycorrhizal fungi and root endophytes increase root lifespan by protecting against desiccation, pathogen attack, or benefiting plant growth by providing nutrients and water resources. Increased pressure from pathogen attack, saprophytic fungi, and herbivores decrease lifespan in *Pathway 3* while increased root respiration, excessive production of reactive oxygen species (ROS) and reactive nitrogen species (RNS) and eventual tissue damage under stressful growing conditions decrease lifespan in *Pathway 4*. Pathways 1 and 4 can be considered intrinsic responses by the plant or root while Pathways 2 and 3 are driven more by changes in beneficial or antagonistic factors that are extrinsic to the plant.

In this framework, we propose a set of four broad pathways through which root lifespan is either reduced or extended along environmental gradients (**Figure 1**). Pathways 1 and 2 represent potential increases in root lifespan through increased allocation of plant resources to absorptive fine roots under favorable growing conditions and increased carbohydrate supply (Pathway 1) or by colonization of roots by beneficial organisms including some mycorrhizal fungi and root endophytes which can provide direct protection from pathogens and saprotrophs or improve whole-plant nutrient balance (Pathway 2). Pathways 3 and 4 reduce lifespan through increased pressure from soil pathogens, saprophytic fungi, and herbivores (Pathway 3) or by physiological stress within the root resulting from poor growing conditions, high respiration, and buildup of free radicals (Pathway 4). Pathways 1 and 4 can generally be considered intrinsic plant responses while 2 and 3 are more extrinsic. Of particular importance will be determining if and where different environmental factors transition from being resource factors benefitting plant health and root lifespan to becoming stress factors impairing root function and reducing lifespan.

Using this framework and evidence from empirical studies, we present a series of conceptual models predicting responses of fine root lifespan to environmental gradients. This review draws on studies covering a range of plant growth forms and biomes and the conceptual models presented here are intended to apply generally to different species and systems. However, a greater number of published studies originate from temperate regions and therefore portions of our discussion and interpretation may be weighted more heavily toward temperate species. We assume that the balance between the four broadly defined pathways in **Figure 1** will determine how root lifespan of a given species will respond to altered environmental conditions. This approach follows many of the same assumptions found in cost-benefit analyses (Eissenstat and Yanai, 1997) and largely discounts the very real possibility that in the face of complex competitive interactions among plants, roots may not always behave in what is predicted to be most optimal (Dybzinski et al., 2011). Though speculative in nature, these first approximations serve as testable hypotheses and provide a starting point from which patterns of fine root lifespan that do and do not fit the models can be interpreted. The conceptual models and discussions presented below are most relevant to the distal, fast-cycling, absorptive portion of the root system that typically represents the first two or three root orders in woody species and is most active in resource uptake (Pregitzer et al., 2002; Xia et al., 2010).

## VARIATION IN FINE ROOT LIFESPAN ALONG ENVIRONMENTAL GRADIENTS

### TEMPERATURE

Most regions of the earth’s surface are expected to experience increases in average temperatures in the coming decades (Stocker et al., 2013). As such, many studies have focused on the effects of rising temperature on ecosystems and belowground processes. Results from these studies have been relatively consistent with most reporting decreased root lifespan with increasing temperatures (Forbes et al., 1997; King et al., 1999; Majdi and Ohrvik, 2004; Leppälammi-Kujansuu et al., 2014), or in some cases, no change (Fitter et al., 1999; Johnson et al., 2006). However, a study by Bai et al. (2012) did observe that constraining increased temperature to daylight hours (i.e., equal temperatures at night between control and treatment groups) induced a slight increase in root lifespan. In the same study, results became mixed between the three different warming treatments, indicating potentially complex feedbacks between root lifespan and temperature, which only partly constrained growth and aboveground productivity in that system. Similar to the majority of experimental manipulations, field observations across sites or through time also indicate that lifespan likely decreases with increasing temperature (Hendrick and Pregitzer, 1993; Watson et al., 2000; Tierney et al., 2003; Kitajima et al., 2010), though in some cases changes in temperature may be secondary to other site factors (Burton et al., 2000).

In addition to broad increases in average temperature, increased temperature variability also appears to decrease root lifespan. A study by Jones et al. (2003) created artificial forest gaps which increased local temperature variability along the gap edges and led to reduced root lifespan. Tierney et al. (2001) and Gaul et al. (2008) also found that by removing snow, which increased temperature fluctuations albeit at lower temperatures, root lifespan decreased as root mortality increased later in the season. Still, shortened lifespan from snow removal experiments may also be interpreted as a result of colder temperatures, freezing stress, or stress from multiple freeze–thaw cycles.

**FIGURE 2:**
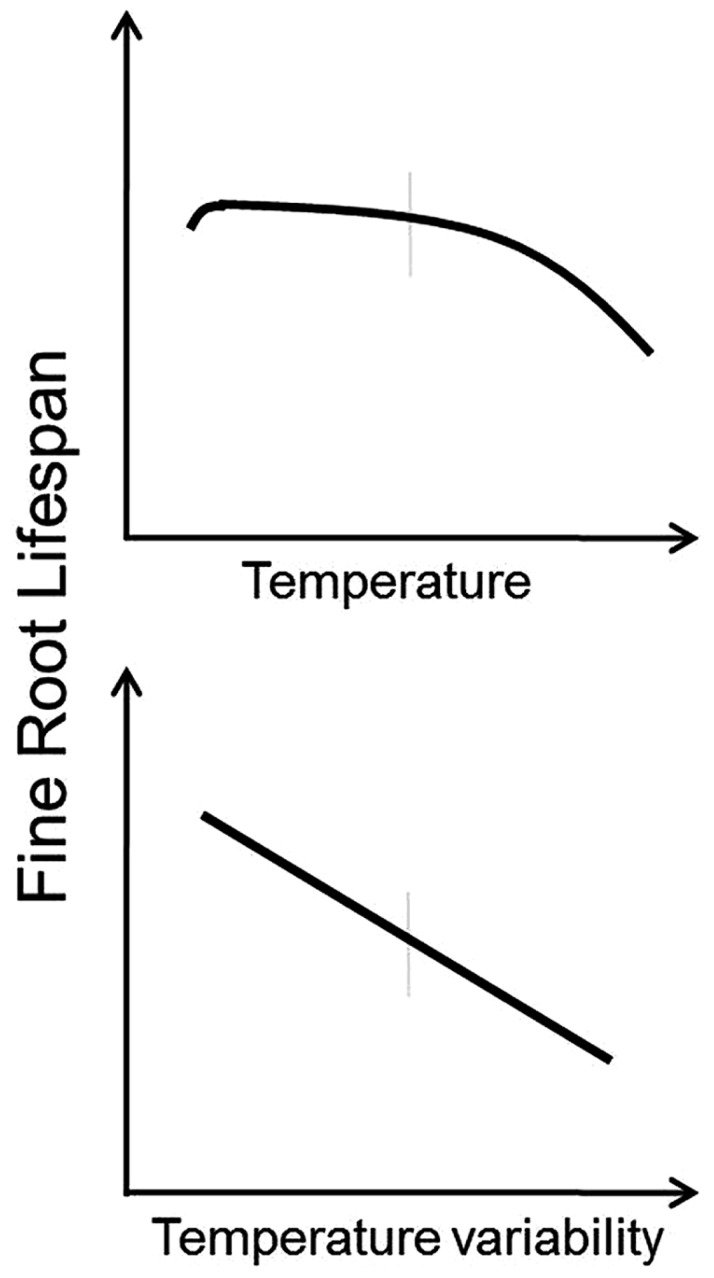
**Conceptual models indicating predicted responses of fine root lifespan to increased temperature (top panel) and increased temperature variability (bottom panel).** Vertical gray bars indicate “average” conditions experienced by a given species across its native range.

Based on these observations, it appears that under most circumstances root lifespan will decrease with increasing temperature and with increasing temperature variability (**Figure 2**). Roots growing at lower temperatures tend to have lower respiration rates (Burton et al., 2002) and increasing mortality and decreased root lifespan at higher temperatures may be the result of increased metabolic activity, buildup of free radicals, and faster root aging (Pathway 4 in **Figure 1**). Furthermore, increased temperature variability may limit the ability of roots to acclimate to increased temperatures and exacerbate the effects of temperature change. However, there are likely to be complex interactions between temperature and plant productivity, nutrient mineralization rates, and water use (see discussions below).

### WATER

The availability of water acts as a fundamental constraint on plant growth and determines the structure of many plant communities. Indeed, limitations in water have led to some of the more distinct patterns of root production and lifespan. For example, “rain roots” in some desert species proliferate rapidly following precipitation events only to be shed relatively quickly as soils again dry out (Nobel et al., 1990). Outside of desert ecosystems, water availability is still an important factor that may have both a positive and negative effects on root lifespan (see Eissenstat et al., 2013 and references therein).

**FIGURE 3:**
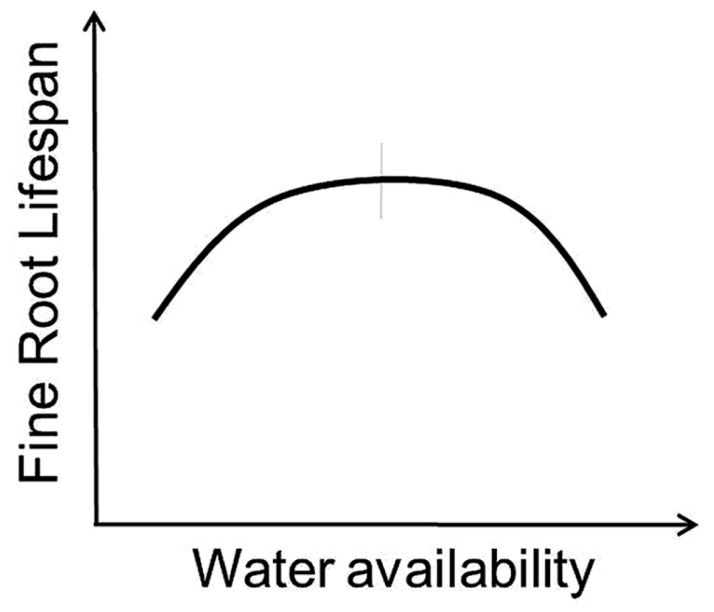
**Conceptual model indicates predicted response of fine root lifespan to altered water availability.** Vertical gray bar indicates “average” conditions experienced by a given species across its native range.

Variation in the response of root lifespan to changes in water availability likely depends on whether water strongly limits root or whole-plant growth. On one side, adding water and alleviating drought should increase whole-plant productivity and increase root lifespan (Mainiero and Kazda, 2006; Misson et al., 2006; Peek et al., 2006; Meier and Leuschner, 2008). Higher precipitation has also been associated with increased root longevity in some tropical systems as root production and lifespan tends to increase during wet seasons and decrease during dry periods (Green et al., 2005). However, additional water applied to an environment that already has adequate moisture may in fact reduce lifespan (Leppälammi-Kujansuu et al., 2014) as the frequency of anoxic conditions elevates root stress and pressures from external factors including soil pathogens and saprophytic fungi increase (**Figures 1** and **3**).

Importantly, as water availability can vary dramatically across a site with changes in soil characteristics and topographic position, so too might the response of individual roots to changes in soil moisture. Perhaps reflecting within-site variability, root responses to patch-level water additions may differ from those at the whole plot level. Pregitzer et al. (1993) found that by adding water to small patches of soil median fine root lifespan was increased by 50 to 75% even though background conditions were not strongly limiting. Here, patch-level additions may have prevented local root stress from soil drying but still avoided stress from anoxic conditions or increased pressure from pathogens.

Hydraulic redistribution may also play a role in mediating responses of fine roots to drought. In a study by Bauerle et al. (2008), lifespan of fine roots growing in dry soil was reduced in the absence of hydraulic redistribution. However, when conditions allowed for redistribution of water from wetter soil to dryer soil, the lifespan of roots in dry soil was maintained at the same level as roots under non-stressed conditions. Overall, accounting for differences in treatment (whole-plot vs. patch) and local soil conditions will help to better understand potential differences in root responses to altered soil moisture.

### NITROGEN

The effect of N addition on root dynamics has perhaps received more attention than any other environmental factor, yet, little broad consensus has been found. Studies directly observing root dynamics have variably reported increased, no change, and decreased fine root lifespan (see Brassard et al., 2009; Chen and Brassard, 2013; Eissenstat et al., 2013). Additionally, a number of conceptual models have been proposed to understand and simplify the responses of root production, mortality, turnover, and lifespan to varying N availability (Hendricks et al., 1993; Burton et al., 2000; Nadelhoffer, 2000). Given the relatively large number of studies, why have few consistent patterns emerged? Variable methodology, intrinsic species differences, and complex interactions with the soil environment have all likely contributed to the diversity of responses observed.

**FIGURE 4:**
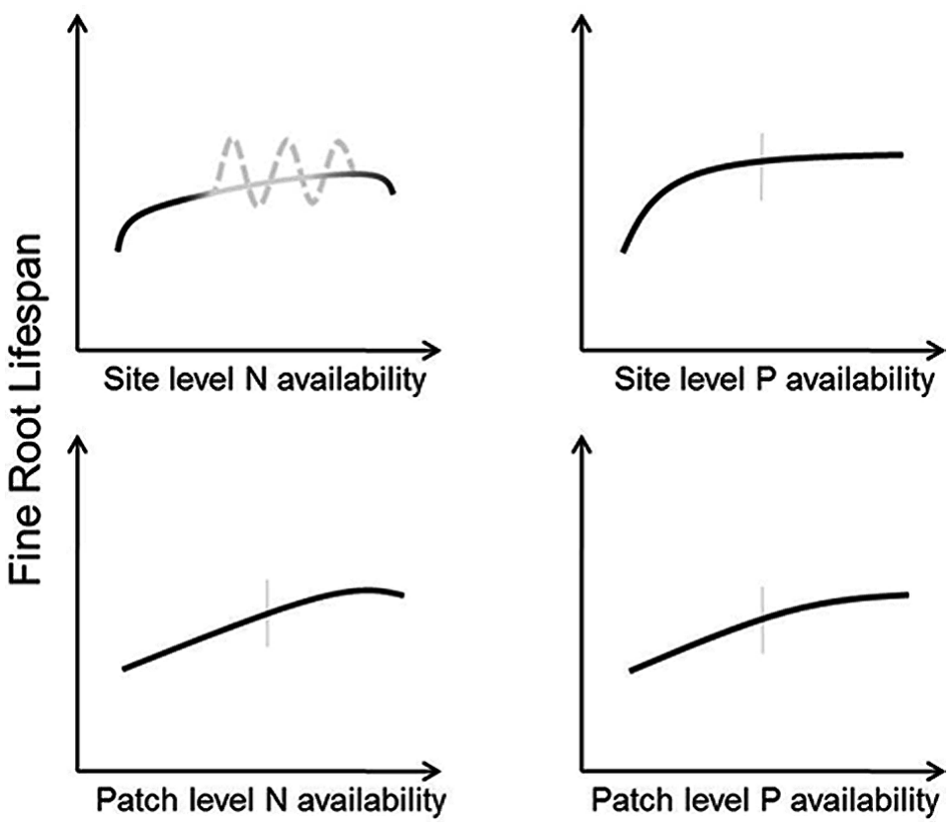
**Conceptual models indicating predicted responses of fine root lifespan to altered N (left panels) and P (right panels) at the site level (top panels) and patch level (bottom panels).** Vertical gray bars indicate “average” conditions experienced by a given species across its native range. The gray portions of the predicted model for root responses to site level N availability indicate uncertainty regarding site specific effects. Here, the solid line is what might be expected where all conditions of species differences, variable methodology, and soil buffering capacity are held constant whereas the dashed line acknowledges that lifespan could both increase or decrease in response to altered N across a wide range of N availability.

Methodological differences in the amount, form, and scale of application can lead to variable results with N fertilization. Interestingly, studies directly testing multiple levels of N addition have found positive (Kern et al., 2004), or negative (Johnson et al., 2000) effects regardless of fertilization amount indicating that factors other than N amount are responsible for cross-site differences in root lifespan. There is, however, some indication that organic and mineral forms of N can elicit different effects on root lifespan (Baldi et al., 2010). Furthermore, scale of N addition may also lead to differential root lifespan changes. Patch-level additions that create fertile microsites to mimic the heterogeneous soil environment increased fine root lifespan in some species (Pregitzer et al., 1993; Adams et al., 2013). These results may be interpreted as the result of plants allocating additional C-resources to roots with greater nutrient uptake so long as plant nutrient demand exceeds resource uptake (**Figure 4**). By contrast, whole-plot level N additions may decrease root lifespan if the treatments saturate plant demand and result in damage to root tissues as the costs of N uptake and storage (increased respiration and the generation of free radicals; Thompson et al., 1987; Bloom et al., 1992) outweigh the benefits.

Intrinsic species differences may also have a role to play in defining fine root responses to increases in N availability (Burton et al., 2000). Though relatively few studies have been able to assess changes in species-specific root lifespan to increased soil N within the same site, recent results have highlighted variable and sometimes contradictory responses among species. Using a common garden study, Adams et al. (2013) found increased root lifespan for two tree species (*Populus tremuloides* and *Acer negundo*) but no change in lifespan for two others (*Sassafras albidum* and *Liriodendron tulipifera*) following patch additions of N. After whole-plot fertilizations, M. Coleman (personal communication) observed a decrease in lifespan for *Pinus taeda* and a slight increase in lifespan for *Populus deltoids*. These mixed responses echo those found by Thomas et al. (2009), where long-term N deposition resulted in both increased and decreased productivity and mortality among 24 tree species which highlights the need for better understanding of inherent differences in species responses to environmental gradients.

The addition of N in terrestrial ecosystems also interacts with the biotic and abiotic soil environment, thus, its impact on root growth and lifespan may go beyond simple alterations in mineral nutrition (Frey et al., 2004; Ramirez et al., 2010; Marschner, 2011). Smithwick et al. (2013) recently proposed that much of the variation in root responses to N additions could be due to inherent differences in the soil capacity to buffer deleterious effects of excessive soil N. For example, chronic N deposition can progressively leach base cations and lower soil pH leading to increased mobility of Al; causing negative effects on root tissue health and the uptake of essential micronutrients (e.g., calcium; Kochian et al., 2005; Marschner, 2011). Therefore, soil capacity to buffer changes in soil acidity may dictate the short-term and long-term consequences for root lifespan following N additions, particularly under N-rich conditions, which is becoming more common in some regions following chronic atmospheric N deposition.

Given these potential complications, consistent predictions of root responses to changes in N availability at the whole-plot level are difficult. However, if experimental methodologies and soil characteristics are held constant root lifespan should increase with increasing N until availability begins to exceed demand, at which point lifespan should decrease due to deleterious effects of N and increased soil acidity (**Figure 4**). However, it is also possible that lifespan may increase with decreasing N availability in some circumstances, particularly in cases where N exists in mobile forms (e.g., nitrate-N). Here, old roots may be retained to minimize resource loss through turnover of root tissue (Chapin, 1980), and a balance must be struck between retaining elemental resources in root tissues and declining uptake rates with root age (Bouma et al., 2001; Volder et al., 2005). More precise predictions of N effects on root lifespan will likely require a better grasp of the interactions between N additions and the physical, chemical, and biological components in the soil. Therefore, future studies measuring responses in fine root lifespan will benefit from more complete assessments of site conditions and soil buffering capacity.

### PHOSPHORUS

Much like extreme water limitation, ecosystems that are severely limited in available P have produced some of the most extraordinary adaptations in root morphology (Lambers et al., 2008). Beyond increases in total surface area, adaptations to low-P conditions may also involve changes to root physiology and morphology, leading to alterations in root lifespan. For example, under low-P conditions thinner roots may be produced to increase absorptive area per unit mass. Previous studies have consistently reported that thinner roots, both within and across species, tend to have shorter lifespans than thicker roots (Wells and Eissenstat, 2001; Baddeley and Watson, 2005; McCormack et al., 2012). Therefore, shifting allocation of resources to thinner roots under low-P conditions is likely to result in decreased root lifespan. Evidence from agricultural studies also indicates that under low-P conditions plants may increase the production of root cortical aerenchyma which enables the root to maintain larger root diameters but reduce the overall root respiration and total root cost (Postma and Lynch, 2011). Here again, constructing less dense (weakly defended) roots such as those with high cortical aerenchyma may lead to reduced root lifespan.

Short lifespan in low-P soils can have potential benefits. Given the limited mobility of P in soil, it may be advantageous to repeatedly build short-lived roots that can explore new volumes of soil whereas long-lived roots will deplete local P reserves and become progressively less efficient at obtaining soil P. Overall, as site or patch P fertility increases, root lifespan should increase until P availability exceeds whole-plant demand which should occur sooner with increases whole-plot P availability than in patches (**Figure 4**). Because excessive P levels rarely occur in nature, root lifespan does not show a drop at relatively high P levels as in the case of excessive N (**Figure 4**). These predictions may be altered by mediations from mycorrhizal fungi, which respond strongly to alterations in soil fertility (Treseder, 2004). It is therefore possible that changes in mycorrhizal colonization in response to available P may even supersede any direct P effects on root lifespan.

### GENERAL PRINCIPLES AND INTERACTIONS AMONG MULTIPLE FACTORS

Given current limitations in observing the belowground component of natural ecosystems, it is often difficult to predict changes in root dynamics in relation to broader changes in climate or fertility. As a first approximation, a few general principles may be drawn (**Table 1**). First, when plant growth is strongly limited by a given factor or resource, increases in that factor should result in increased lifespan (e.g., increasing water to alleviate drought). This occurs as stress is relieved, first at the root level (e.g., avoiding desiccation with severe drought, Pathway 4 in **Figure 1**), and later at the whole-plant level as growing conditions improve, carbohydrate reserves increase, and plants can allocate additional resources to maintain root performance or distribute resources to root symbionts (Pathways 1 and 2 in **Figure 1**). Second, when a given factor or resource is in excess of plant need or demand, further increases should result in a decline in root lifespan (**Figure 1**). This occurs as stress factors at the root level increase (e.g., heat stress and high respiration with temperature, anoxic conditions with excessive water) and whole-plant growth is again impaired. Based on these two assertions the longest fine root lifespan for a given species should be found in locations with average climate levels for a given species and moderate to high, but not excessive fertility levels.

**Table 1:** General principles for predicting responses in root lifespan with changes in key environmental factors: temperature, water availability, nitrogen, and phosphorus.

1. Increases in a factor or resource that strongly limits plant growth will increase fine root lifespan
2. Increases in a factor or resource in excess of plant demand or tolerance will decrease fine root lifespan
3. As multiple environmental factors interact, the likely response of fine root lifespan should be determined by the factor that is most limiting plant growth

In nature, different environmental factors may interactively influence root lifespan. Given the difficulty in disentangling complex effects of multiple variables on fine root dynamics, we propose a third principle defining root lifespan responses to environmental change: as multiple factors interact, changes in that factor which serves as the primary constraint on the plant system should have the greatest effect on fine root lifespan. Furthermore, changes in other factors that subsequently alter the primary factor will affect root lifespan indirectly through the primary factor rather than directly. Consider two ecosystems of similar vegetation, soil, and precipitation but different temperature regimes. Temperature is the apparent factor that varies between the sites, but changes in temperature may also alter plant water use and soil water availability. Therefore, the two sites may also differ in their water availability which, depending on site conditions, can ultimately be of greater importance. Further complicating matters is the fact that both changes in temperature and water availability will alter decomposition and nutrient mineralization rates within the ecosystem. As such, the response to a simple temperature gradient may be governed by interactions among temperature, water availability, and nutrient availability. In many cases, the ultimate constraining factor may not temperature per se but other factors such as soil moisture as has been shown in multi-factor studies in the temperate steppe of Northern China (Niu et al., 2008).

Overall, when considering how different environmental factors interact, it will be important to identify the major constraints within an ecosystem and then to determine how changes in other factors will impact the dominant constraining factor. Depending on local site conditions, some factors may stand out as more important than others, even upon occasions where they are not the primary factor being observed or manipulated. Furthermore, different strategies may be employed by some plants to counterbalance some environmental factors, particularly in resource limiting situations. Where elemental resources are limiting it may be advantageous to extend root lifespan thereby minimizing resource loss through root tissue turnover (Chapin, 1980). Future work identifying the capacity of different species to resorb elemental resources from aging root tissues will improve understanding of the role that adjustments in root lifespan may play in nutrient limiting environments.

## COMPLICATIONS AND OPPORTUNITIES FOR UNDERSTANDING FINE ROOT LIFESPAN

### ROLE OF ROOT TRAITS AND MYCORRHIZAL FUNGI IN DETERMINING PLASTICITY OF ROOT LIFESPAN

Key to understanding variation in root lifespan within a species is to determine which species or what types of species are likely to be most plastic when facing changes in temperature, water availability, and soil fertility. The degree to which environmental factors impact root lifespan may be related to root morphology and chemistry, whole-plant growth strategies (e.g., fast- vs. slow-growing species), or the type and intensity of mycorrhizal association. While direct comparisons here are few, some evidence of systematic differences among species has emerged. Adams et al. (2013) found that in a common garden the root lifespan of two fine-rooted species was significantly altered by patch additions of N fertilizer whereas root lifespan in two coarse-rooted species was unaffected. This is consistent with other reports that fine-rooted species respond more markedly to nutrient rich patches than coarse-rooted species (Eissenstat, 1991; Fitter, 1994). Overall, we hypothesize that species with thinner roots and/or lower root construction costs, and are either non-mycorrhizal or facultative mycotrophs should exhibit the greater plasticity in their root lifespan. In contrast, species with coarse roots, higher construction costs, and obligate mycotrophs will likely be less responsive to changes in different environmental factors (Box 1). Given the wide range of cross-species variation in root morphology, particularly root diameter (Chen et al., 2013), and many other traits, future studies focusing on systematic trait variation will likely to be critical in identifying which species express the most variation in root lifespan along environmental gradients (**Table 2**).

**Table 2:** Key questions for future research.

**FIGURE 5:**
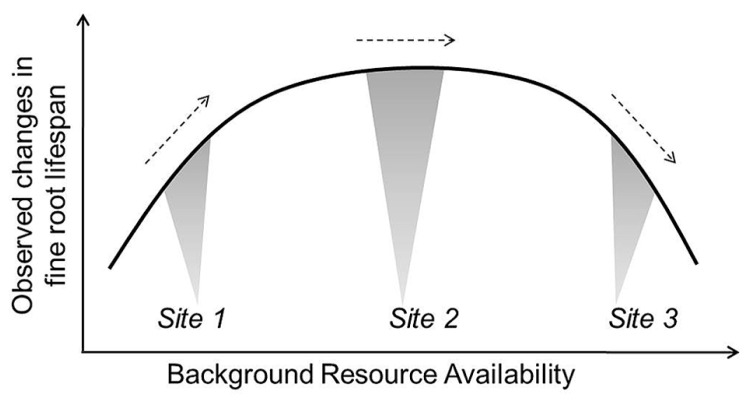
**Conceptual example of how equivalent additions of the same resource (e.g., irrigation or N fertilization) could result in observed increases, decreases, and no change in fine root lifespan depending on background resource availability or other site characteristics.** Site 2 represents *average* background resource availability and soil conditions. Site 1 is low in resource availability and would see an increase in root lifespan with an increase in a given resource while lifespan decreases in Site 3 where resource availability has exceeded plant demand and the buffering capacity of soils (e.g., anoxia or decreasing soil pH).

1. What species or types of species are likely to express greater plasticity in response to different environmental factors?
2. How will the shapes or even the directions of the curves presented in **Figures 2**–**5** differ among biomes?
3. Can numerical models be used to clarify complex relationships between root dynamics and ecosystem processes along environmental gradients?

**Box 1. Hypothesized plasticity of different species in response to environmental variation based on the type of fine root architecture (e.g., fine roots with larger or smaller diameter, tissue density) or degree of mycotrophy expressed.**

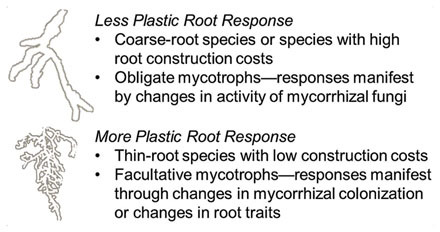

Currently, the role that mycorrhizal fungi play in modulating root responses to environmental variability remains largely unknown. A wide taxonomic array of fungi form intimate relationships with the majority of terrestrial plant roots (Smith and Read, 2008), and similar to roots, determining the responses of mycorrhizal fungi to changing climate and soil conditions is of great importance but fraught with difficulty (Read and Perez-Moreno, 2003; Treseder, 2004; Phillips et al., 2013). A review by Chen and Brassard (2013) found no support for mycorrhizal influence on root lifespan. However, reports of this type are rare and this assessment may change as more studies emerge. Given their large taxonomic range and functional diversity, different species or types of mycorrhizal fungi may induce different effects on plant roots. For example, highly melanized fungi were found to increase root persistence and retard decomposition which together may lengthen functional root lifespan (Langley et al., 2006; Fernandez et al., 2013). Conversely, colonization by other mycorrhizal fungi can increase N content of roots, potentially leading to more frequent grazing by soil herbivores and accelerated decomposition (Langley and Hungate, 2003; Koide et al., 2011).

While the direct effects of fungal colonization on root lifespan remain elusive, several studies have found that different mycorrhizal fungi can confer resistance to various environmental stress factors including drought (Parke et al., 1983; Wu and Xia, 2006), temperature extremes (Bunn et al., 2009; Zhu et al., 2010), salt stress (Ruiz-Lozano et al., 1996), and metal toxicity (Godbold et al., 1998; Rivera-Becerril et al., 2002). Therefore, in some cases colonization by mycorrhizal fungi enable stress tolerance or avoidance to individual roots and may benefit roots by improving whole-plant nutritional status or water balance (**Figure 1**). Regardless, the specific effects are likely to depend on the strength of the stress as well as the fungal and plant species involved.

### SOIL AS A COMPLEX BUFFER TO ENVIRONMENTAL VARIABILITY

In the previous sections, we have alluded to the role that soils play in modulating root responses to environmental factors. Highly complex, soils have the capacity to enhance or buffer plant responses to changes in climate and soil fertility through varied physical and chemical properties. For example, though the frequency and intensity of water stress is certainly defined in part by precipitation, physical properties of soil largely determine the total water storage capacity of an ecosystem and the amount of water available for plant uptake (Jury and Horton, 2004; Brady and Weil, 2010). As a result, even under identical patterns of rainfall, two sites can have vastly different patterns of water availability and stress if one is dominated by clay soils and another sandy. Therefore, when predicting precipitation effects on root lifespan, one should consider the simultaneous effects of soil texture on water retention and availability. Also worth considering are soil bulk density and soil color (which impacts the albedo of an ecosystem), which can modulate the temperature within an ecosystem and the rate at which soils warm and cool during spring and fall and subsequently influence root phenology, microbial activity, and water availability.

Beyond primarily physical properties, soils also play a large role mediating elemental cycles in a system. Interactions between soil cation exchange capacity, acidity, and the movement of anions and cations can be vital in determining root responses to environmental factors. The effects of soil exchange and buffering capacities may indeed be key determinates of root responses to N fertilization (Smithwick et al., 2013). More attention to these and other aspects of soil chemistry that regulate macro- and micro-nutrient availability as well as concentrations of potentially toxic elements (e.g., Al^3^^+^) are likely to greatly improve our understanding of root responses to environmental factors at scales ranging from individual roots and species to whole-ecosystems and communities.

### IF I AM A MODELER, WHAT SHOULD I DO?

Increases in computational power and improvements in model skill have allowed some models to include explicit relationships between root lifespan and other parameters within the modeled environment (e.g., climate, soil fertility). For example, root lifespan/turnover varies as a function of annual N mineralization in PnET-CN (Aber et al., 1997; Ollinger et al., 2002), as a function of soil temperature in ED2 (Medvigy et al., 2009), and temperature + water stress in the FORCENT model (Parton et al., 2010). However, given the large uncertainty of predicted relationships between root dynamics and environmental variables, it is still unclear which factors to include. Here, models should play a role in testing potential relationships between environmental factors and root lifespan. By determining *likely* responses through sensitivity analyses, models can help to (1) focus field efforts on the relationships with greatest uncertainty and impact on model outputs (LeBauer et al., 2013; Wang et al., 2013) and (2) focus studies to test root responses across regions of greatest interest (e.g., nutrient addition studies in low fertility vs. high fertility sites).

Based on current evidence, it seems reasonable for models to assume decreasing lifespan with increased temperature and water stress, though the specific rate of change in lifespan with a unit increase in temperature or water is more difficult to set. Given the often contradictory reports in the literature, approaches modulating root lifespan based on N mineralization rates may be more tenuous when applied beyond the original ecosystems where the turnover-N mineralization relationships are developed. However, this too is likely a useful approach that can be used to further test relationships between root dynamics, background site conditions, and nutrient additions. Future efforts may link root lifespan-N relationships with more complex functions including additional soil factors (e.g., pH, soil buffering capacity) pending development of more clear relationships among them. In all cases, more work is needed to observe and define *average* conditions so that we can better appreciate when increases or decreases in a given environmental factor will move fine roots toward or away from their maximum lifespan (**Figure 5**). Additionally, modeling work defining which factors are likely to be most limiting or constraining for a given site may help to predict changes in root dynamics in response to multiple, interacting factors.

### MOVING FORWARD

The conceptual models presented in **Figures 2**–**5** represent testable hypotheses. Each model is based on empirical data when possible along with a simple logical framework (**Figure 1**). Though simple in their presentation here, patterns of root responses to environmental changes are likely to be complex and contingent at least in part on prevailing climatic conditions across biomes, specific climate and background soil conditions within a site, and interactions with mycorrhizal fungi. While it is likely that further study may amend or even change the specific predictions entirely, these conceptual models offer a reference against which field experiments and modeling work can be compared. In near term, we may test the predictions from these conceptual models at selected, individual sites and manipulative studies to gain a baseline understanding of root lifespan–environment relationship. However, observations of fine root lifespan along natural environmental gradients across sites are also much needed and may provide the most useful information regarding within species variation. Efforts should also be made to better appreciate fundamental differences in plasticity among different species in response to environmental factors so that regions/species likely to have the greatest variation and the largest uncertainty can be expressly targeted (**Table 2**).

Over the long run, the goal should be to rigorously test predictions across a range of plant species and biomes. Additionally, building a greater research portfolio investigating multifactor effects on fine root lifespan and determining under which conditions each factor will be the dominant driver of altered fine root lifespan will be a tremendous improvement predicting and modeling root dynamics. Given the difficulty and long-term (>5 year) commitment required to observe and assess root dynamics, we may not be able to rapidly address many of the recommendations. However, linking the currently disparate observations of root dynamics spread across the globe into more cohesive networks with greater similarity in methodology will likely serve as an efficient path toward greater predictability and deeper understanding of fine root and ecosystem processes.

## Conflict of Interest Statement

The authors declare that the research was conducted in the absence of any commercial or financial relationships that could be construed as a potential conflict of interest.
